# F7. SEX DIFFERENCES IN THE ASSOCIATION BETWEEN SELF-REPORTS OF CHILDHOOD ADVERSITIES AND SCHIZOTYPAL PERSONALITY TRAITS

**DOI:** 10.1093/schbul/sby017.538

**Published:** 2018-04-01

**Authors:** Diamantis Toutountzidis, Tim Gale, Karen Irvine, Shivani Sharma, Keith Laws

**Affiliations:** University of Hertfordshire

## Abstract

**Background:**

While it has been repeatedly documented that people with schizophrenia report higher levels of adverse events in childhood (emotional, physical and sexual abuse), this has not been extensively examined in healthy individuals who score highly on schizotypal personality traits. The continuum hypothesis of psychosis and schizophrenia suggests it is important to assess the relationship in those who are healthy but who experience some psychotic-like symptoms. Of course, it is problematic to rely upon the veracity of events that anyone might recall from their childhood, but this is likely to be compounded by the presence of well-documented memory and executive problems, as well as symptoms such as delusional thinking, in some adults with psychosis. One advantage of examining healthy participants is that recall is not affected by the condition itself or memory- and executive-function problems. As there is evidence that the expression of psychotic disorders differ between males and females, the etiological mechanisms and pathways to the development and experience of psychotic symptoms may equally differ. Indeed, sex differences in the association between childhood trauma and psychotic symptoms have been noted. The aim of this present study was to investigate any links between childhood trauma and psychotic-like symptoms in healthy individuals. Based on previous research the expectation is that associations will be found between self-reports of childhood trauma and schizotypal personality traits. These associations would be expected to differ between males and females.

**Methods:**

The sample consisted of 320 participants (221 females, 99 males) with a mean age of 28.24 (SD 12.76). Childhood traumatic events were assessed by three sub-scales (Physical Punishment; Emotional Abuse; and Sexual Events) of the Early Trauma Inventory Self Report-Short Form (ETISR-SF; Bremner et al., 2007). Schizotypal personality traits were assessed using the Five Factor Schizotypal Inventory (FFSI; Edmundson et al., 2011). This consists of nine subscales (Interpersonal Suspiciousness; Social Anhedonia; Social Isolation and Withdrawal; Physical Anhedonia; Social Anxiousness; Social Discomfort; Odd and Eccentric; Aberrant Ideas; and Aberrant Perceptions) which were constructed as schizotypic variants of respective facets of the five factor personality model.

**Results:**

The relationship between childhood trauma and schizotypy was examined using Spearman’s bivariate correlation analyses. Males showed significant positive correlations (ranging from .28 to .39) between Emotional Abuse and seven out of nine schizotypal sub-scales. The other two childhood trauma scales were not associated with any schizotypal sub-scales in males.

Females showed significant positive correlations (ranging from .19 to .34) between Physical Punishment and eight out of nine schizotypal sub-scales. Additionally, females showed significant positive correlations (ranging from .26 to .35) between Emotional Abuse and all schizotypal sub-scales. Sexual Events positively correlated with two schizotypal sub-scales (Aberrant Ideas and Aberrant Perceptions) in females.

**Discussion:**

From the results of this study it appears that emotional abuse was linked to the expression of psychotic-like symptoms in both sexes across a wide array of symptomatology. Therefore, any effect of emotional abuse should not be considered sex or symptom specific. By contrast, physical abuse appeared to be sex specific – affecting only females – but not symptom specific. Finally, sexual abuse appeared to have a specific link with disorganized thoughts and hallucination-like experiences in females.

